# Two-dimensional ferromagnetic superlattices

**DOI:** 10.1093/nsr/nwz205

**Published:** 2019-12-16

**Authors:** Shanshan Liu, Ke Yang, Wenqing Liu, Enze Zhang, Zihan Li, Xiaoqian Zhang, Zhiming Liao, Wen Zhang, Jiabao Sun, Yunkun Yang, Han Gao, Ce Huang, Linfeng Ai, Ping Kwan Johnny Wong, Andrew Thye Shen Wee, Alpha T N’Diaye, Simon A Morton, Xufeng Kou, Jin Zou, Yongbing Xu, Hua Wu, Faxian Xiu

**Affiliations:** 1 State Key Laboratory of Surface Physics and Department of Physics, Fudan University, Shanghai 200433, China; 2 Institute for Nanoelectronic Devices and Quantum Computing, Fudan University, Shanghai 200433, China; 3 Laboratory for Computational Physical Sciences (MOE), Fudan University, Shanghai 200433, China; 4 Department of Electronic Engineering, Royal Holloway University of London, Egham TW20 0EX, UK; 5 School of Electronic Science and Engineering, Nanjing University, Nanjing 210093, China; 6 Materials Engineering, The University of Queensland, Brisbane QLD 4072, Australia; 7 Department of Physics, National University of Singapore, Singapore 117542, Singapore; 8 Centre for Advanced 2D Materials and Graphene Research Centre, National University of Singapore, Singapore 117546, Singapore; 9 Lawrence Berkeley National Laboratory, Berkeley, CA 94720, USA; 10 School of Information Science and Technology, ShanghaiTech University, Shanghai 201210, China; 11 Centre for Microscopy and Microanalysis, The University of Queensland, Brisbane QLD 4072, Australia; 12 Collaborative Innovation Center of Advanced Microstructures, Nanjing 210093, China

**Keywords:** 2D ferromagnetic material, room temperature, 2-inch Fe_3_GeTe_2_ film wafers, proximity effect, (Fe_3_GeTe_2_/CrSb)_n_ superlattice

## Abstract

Mechanically exfoliated two-dimensional ferromagnetic materials (2D FMs) possess long-range ferromagnetic order and topologically nontrivial skyrmions in few layers. However, because of the dimensionality effect, such few-layer systems usually exhibit much lower Curie temperature (*T*_C_) compared to their bulk counterparts. It is therefore of great interest to explore effective approaches to enhance their *T*_C_, particularly in wafer-scale for practical applications. Here, we report an interfacial proximity-induced high-*T*_C_ 2D FM Fe_3_GeTe_2_ (FGT) via A-type antiferromagnetic material CrSb (CS) which strongly couples to FGT. A superlattice structure of (FGT/CS)_n_, where *n* stands for the period of FGT/CS heterostructure, has been successfully produced with sharp interfaces by molecular-beam epitaxy on 2-inch wafers. By performing elemental specific X-ray magnetic circular dichroism (XMCD) measurements, we have unequivocally discovered that *T*_C_ of 4-layer Fe_3_GeTe_2_ can be significantly enhanced from 140 K to 230 K because of the interfacial ferromagnetic coupling. Meanwhile, an inverse proximity effect occurs in the FGT/CS interface, driving the interfacial antiferromagnetic CrSb into a ferrimagnetic state as evidenced by double-switching behavior in hysteresis loops and the XMCD spectra. Density functional theory calculations show that the Fe-Te/Cr-Sb interface is strongly FM coupled and doping of the spin-polarized electrons by the interfacial Cr layer gives rise to the *T*_C_ enhancement of the Fe_3_GeTe_2_ films, in accordance with our XMCD measurements. Strikingly, by introducing rich Fe in a 4-layer FGT/CS superlattice, *T*_C_ can be further enhanced to near room temperature. Our results provide a feasible approach for enhancing the magnetic order of few-layer 2D FMs in wafer-scale and render opportunities for realizing realistic ultra-thin spintronic devices.

## Teaser

Utilizing proximity effect, we realize an effective and reliable approach to accomplish near-room-temperature (286.7 K) ferromagnetic order and double switching behavior in wafer-scale 4-layer-Fe_3_GeTe_2_/1.6-nm-CrSb superlattice.

## INTRODUCTION

Two-dimensional (2D) systems involving various functionalities are a central topic in condensed matter physics. Since the discovery of graphene [1,2], the 2D material family has been widely explored in semiconductors [3,4], superconductors [5,6], and ferromagnetic materials (FMs) [7–12]. In particular, spintronic devices based on 2D FMs have attracted significant attention, for example, magnon-assisted tunneling and giant tunneling magnetoresistances were found to possess multiple magnetic states in CrX_3_ (X = Br and I)-based junctions [13–17]. In 2D FMs, the perpendicular magnetic anisotropy that is partially contributed by spin-orbit coupling plays a more essential role in magnetic order as the thickness reduces [8,11]. Theoretically, because of strong spin-orbit coupling and broken inversion symmetry, Dzyaloshinskii-Moriya interactions [18,19] can provide topological magnetic textures, thus inducing skyrmions. Using Lorentz transmission electron microscopy (TEM), at low temperatures Néel-type skyrmions (magnetic bubbles) have been observed in Fe_3_GeTe_2_ (Cr_2_Ge_2_Te_6_) with controllable transitions between skyrmions and magnetic domains [20,21]. However, one unprecedented challenge still exists, that is the suppressed Curie temperature (*T*_C_) as the thickness of 2D FMs decreases [7,11]; this is ascribed to the dimensionality effect of the competing perpendicular magnetic anisotropy energy with thermal fluctuations [11,22]. Modulation of the ferromagnetic properties in few-layer 2D FMs, such as enhancing *T*_C_ or the control of the coercive field (*H*_C_), provides a route towards realistic spintronic applications using 2D FMs.

Recent studies have unveiled the gate-controlled ferromagnetic order in 2D FM nanoflakes. As an example, the ferromagnetic parameters of *T*_C_ and *H*_C_ for monolayer CrI_3_ can be tuned via *h*-BN gating [23,24], and bilayer CrI_3_ exhibits a reversible transition between antiferromagnetic (AF) and FM states [25]. Compared to other 2D FMs, Fe_3_GeTe_2_ is more stable among the recently explored 2D FMs; and by changing the Fe composition [9,26] and applying ionic-liquid gating [11], the ferromagnetism of Fe_3_GeTe_2_ can be modulated. Complementary to these doping and gating techniques, the proximity effect can induce a stable ferromagnetic order through interface coupling [27,28] and avoid the inconvenience of using dielectric gates for the transient FM states. For example, in Bi_2_Se_3_/EuS heterostructures, Bi_2_Se_3_ possesses room-temperature ferromagnetism which is far above the intrinsic *T*_C_ of EuS (17 K) as a result of large spin-orbit coupling [29]; the quantum anomalous Hall effect in graphene has been proposed by proximity coupling to an antiferromagnetic insulator [30]. Except for the FM-induced interfacial magnetism [29,31,32], recent experimental observations suggest that the interplay between antiferromagnetic CrSb and ferromagnetic topological insulators can dramatically enhance the magnetic order in topological insulators with the interfacial spin texture modulation [33]. Such a practicable proximity effect potentially could be applied to control the ferromagnetism of 2D FMs.

Here, we report control of the ferromagnetic order in 2D wafer-scale Fe_3_GeTe_2_ films via the proximity effect using a molecular-beam epitaxy (MBE) growth technique. It is found that the *T*_C_ of Fe_3_GeTe_2_ films reduces with decreasing the thickness, i.e. 220 K for the bulk, 140.3 K for 4-layer and 138.4 K for 2-layer Fe_3_GeTe_2_. By producing FM/AF-structured (FGT/CS)_n_ superlattices with clean interfaces, we find that the *T*_C_ of the 4-layer Fe_3_GeTe_2_ can be monotonously enhanced from 140.3 K (*n* = 0), to 206.3 (*n* = 3) and finally to 230.9 K (*n* = 10), driven by long-range interfacial exchange coupling. Simultaneously, the proximity effect induces a double-switching behavior that gradually smears out as the temperature increases beyond 55 K. By performing temperature-dependent X-ray magnetic circular dichroism (XMCD), we prove that the enhanced *T*_C_ originates from the superlattice Fe_3_GeTe_2_ regions and the double-switching phenomenon stems from the interfacial ferrimagnetic CrSb. In line with these experimental findings, our density functional theory (DFT) calculations demonstrate that the doping of the spin-polarized electrons by the interfacial magnetic Cr layer favors the *T*_C_ enhancement of the Fe_3_GeTe_2_ films rather than the interfacial strain effect, and that the interfacial Cr layers retain the interlayer AF coupling but have a net magnetic moment. Furthermore, by designing 4-layer Fe-rich Fe_3+x_GeTe_2_ and ∼1.6 nm CrSb with the same superlattice structure, we have accomplished the highest *T*_C_ of 286.7 K in (Fe_3+x_GeTe_2_/CrSb)_3_ superlattice, which approaches room temperature with a stable ferromagnetic order over 12 months.

## RESULTS AND DISCUSSION

**Figure 1.**
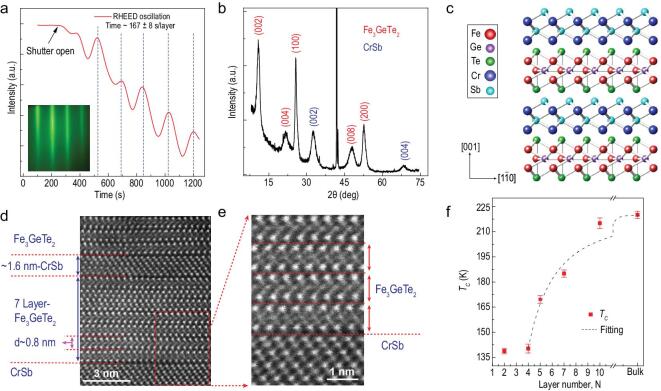
Thin-film growth and characterizations. (a) RHEED oscillations of 2D ferromagnetic Fe_3_GeTe_2_ films. The layer-by-layer epitaxial mode can be verified by the periodic RHEED intensity oscillations, from which the growth period is determined to be ∼167 ± 8 s per layer. The left inset is a streaky RHEED pattern, suggesting the smooth surface of Fe_3_GeTe_2_. (b) An XRD spectrum of (FGT/CS)_6_ superlattice. Peaks from Fe_3_GeTe_2_ and CrSb are marked in red and blue, respectively. The epitaxial orientations of Fe_3_GeTe_2_ and CrSb are ascribed to be along 〈002〉 and 〈002〉, respectively. (c) A schematic geometry of FGT/CS superlattice. Ideally, the *c*-axis of Fe_3_GeTe_2_ and CrSb should be along the same direction; however, experimentally, it has a slight deviation. (d) A cross-section HAADF image of a (FGT/CS)_3_ superlattice, where the thickness of CrSb is estimated to be ∼1.6 nm and Fe_3_GeTe_2_ is 7 layers (∼5.6 nm). (e) A zoom-in HAADF picture. Sharp interfaces between Fe_3_GeTe_2_ and CrSb layers can be clearly distinguished. Note that the Pt layer is deposited during the TEM sample preparation process. (f) Thickness-dependent *T*_C_. As the films become thinner, *T*_C_ has a dramatic drop from 220 K (bulk) to 138.4 K (bilayer) as a result of a strong dimensionality effect. The dashed line is a theoretical fit to the finite-size scaling law.

Layer structured Fe_3_GeTe_2_ has a hexagonal structure with a space group P6_3_/mmc and lattice constants of *a* = *b* = 3.991 Å and *c* = 16.396 Å [34], in which each layer consists of five sublayers with Fe_3_Ge slab sandwiched between two Te layers [9,34]; and the A-type antiferromagnetic (A-AF) CrSb [35] is a NiAs-type structure with a space group of P6_3_/mmc and lattice constants of *a* = *b* = 4.108 Å and *c* = 5.440 Å [36], which serves as an ideal candidate for the epitaxial growth of FM/AF heterostructures. Periodic reflection high-energy electron diffraction (RHEED) intensity oscillations of Fe_3_GeTe_2_ suggest a layer-by-layer growth mode as shown in Fig. 1a. It typically takes 167±8 s to complete 1-layer Fe_3_GeTe_2_ growth, guaranteeing the fine controllability of the film thickness. Detailed growth conditions and high-crystalline characterizations are discussed in detail in the Method section and Supplementary Figs 1–2. Figure 1b displays a typical X-ray diffraction (XRD) spectrum for a (FGT/CS)_6_ superlattice, in which all diffraction peaks can be exclusively indexed as Fe_3_GeTe_2_ and CrSb without any mixtures or new compounds generated, with a schematic geometry of FGT/CS (Fig. 1c). The interlayer distance of Fe_3_GeTe_2_ is half of *c*-axis of ∼0.8 nm (Fig. 1d), consistent with the (002) diffraction peak shown in Fig. 1b and the refinement-XRD value of 8.17 Å [34]. Sharp interfaces between Fe_3_GeTe_2_ and CrSb are shown in the high-angle-annular-dark-field (HAADF) image (Fig. 1d) with the enlarged part displayed in Fig. 1e, further excluding the possibility of element mixing at these interfaces or nearby layers.

We then examined the thickness-dependent magnetic properties of Fe_3_GeTe_2_ films by an anomalous Hall effect (AHE) in the Hall-bar geometry with a size of 1.5 × 2 mm^2^. Even down to 4 layers, the easy axis of Fe_3_GeTe_2_ is still along the *c*-axis (out-of-plane) as *H*_C_ increases with the angle switching from 0° to 90° (see details in Supplementary Fig. 3a). As displayed in Fig. 1f, the Curie temperature (see details about Arrott-plots in Supplementary section 1) shows bulk-like behavior with *T*_C_ of ∼216.4 K when the thickness is above 8 nm (∼10 layers). However, when reducing the thickness, *T*_C_ displays a declined trend and exhibits a dramatic drop below 7-layers. Further reducing to the bilayer, Fe_3_GeTe_2_ retains a ferromagnetic state with *T*_C_ of 138.4 ± 1.6 K. As the Fe_3_GeTe_2_ thickness approaches the 2D limit, the thickness-dependent *T*_C_*(N)* can be described by a universal scaling law [37–39] written as $( {{T_C}( \infty ) - {T_C}( N )} )/{T_C}(\infty ) = {( {({N_0} + 1)/2N} )^\lambda }$, where *T*_C_(∞) denotes the *T*_C_ of the bulk crystal, the critical exponent λ reveals the universality class of the transition, and *N*_0_ is the critical layer number referring to the mean spin-spin interaction range and separating the boundary between the 2D and 3D magnetism. The scaling law function is fitted to the experimental data yielding *N*_0_ ∼ 3.52 ± 0.72 and λ ∼1.79 ± 0.38. The deduced λ also suggests that Fe_3_GeTe_2_ belongs to the Ising-type ferromagnet (note that λ = 1.42 is from the Heisenberg model [40], λ = 1.56 from the Ising model [41], and λ = 1 from mean-field theory [38]). A crossover from 3D to 2D Ising ferromagnetism with the thickness decreasing has been reported in Fe_3_GeTe_2_ nanoflakes [10]. The strong dimensionality effect can be commonly explained by the competition between the magnetic anisotropy energy and prominent thermal fluctuations in thinner samples [7,22].

**Figure 2.**
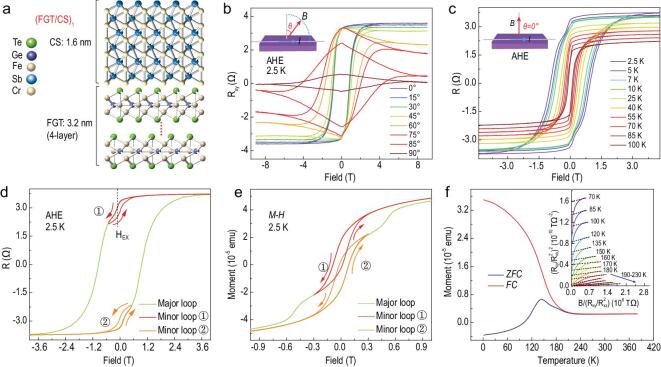
FM/AF interaction induced double-switching behavior in AHE/*M-H* curves and the enhanced *T*_C_ in the (FGT/CS)_3_ superlattice. (a) A schematic structure of one period FGT/CS superlattice that is made up of ∼3.2 nm Fe_3_GeTe_2_ (4-layer) and ∼1.6 nm CrSb. (b) Angle-dependent AHE at 2.5 K with the measurement geometry defined in the inset. The easy axis is determined to be out-of-plane, the same as that of pure Fe_3_GeTe_2_. (c) Temperature-dependent AHE under perpendicular geometry. Inset shows the experimental setup. At 2.5 K, another small switching behavior appears at ∼±0.18 T besides the sharp resistance jump at ∼±0.96 T. Small hysteresis exists when scanning the magnetic field back and forth in a small field region, denoted as minor loops, as displayed in (d). The interaction between Fe_3_GeTe_2_ and CrSb interface is manifested to be ferromagnetic coupling, evidenced by the negative exchange field *H*_EX_ in minor loop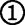. (e) Major and minor *M-H* loops at 2.5 K. (f) *ZFC-FC* data under 200 Oe for (FGT/CS)_3_ superlattice. *T*_C_ is roughly determined to be ∼201 K, comparable to 206.3 ± 1.6 K as deduced by the Arrott-plots in the inset.

To enhance the Curie temperature of Fe_3_GeTe_2_, we geometrically designed FGT/CS superlattices with different periods and thickness. Detailed characterizations of A-AF CrSb under in-plane and out-of-plane magnetic fields are presented in Supplementary Fig. 4, with no sign of ferromagnetism in both measurement geometries. Here, we denote Fe_3_GeTe_2_/CrSb superlattices to be (FGT/CS)_n_, where *n* is the period. Unless specifically mentioned, hereafter, the thickness of Fe_3_GeTe_2_ and CrSb is ∼3.2 nm (4-layer) and ∼1.6 nm, respectively. *n* = 1 stands for the single-period structure of ∼3.2 nm Fe_3_GeTe_2_ and ∼1.6 nm CrSb, as schematically shown in Fig. 2a. To confirm the magnetic anisotropy of (FGT/CS)_3_ superlattice, angle-dependent AHE was performed (Fig. 2b). The easy axis of (FGT/CS)_3_ is still along the out-of-plane direction, sharing the same perpendicular magnetic anisotropy as the pure Fe_3_GeTe_2_. Here, the temperature-dependent AHE was measured under perpendicular geometry (Fig. 2c inset). At low temperatures such as 2.5 K (Fig. 2c), the AHE presents a resistance switching at ±0.97 T accompanied by another weaker switching behavior at a relatively low field. The origin of such property most likely comes from the FM/AF interface [33,42–44] and we define this phenomenon as double-switching behavior. With the temperature increasing, this behavior becomes inconspicuous at ∼55 K, indicating decrease of the interface coupling. Figure 2d presents the switching behavior of the minor loops at low fields. Here, the exchange field (*H*_EX_) is designated to describe the double-switching behavior. Negative *H*_EX_ in minor loop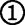 indicates that it is a parallel ferromagnetic coupling between the interfacial Fe_3_GeTe_2_ and CrSb [42]. This double-switching property can also be observed at low-temperature magnetization hysteresis (*M-H* curves, Fig. 2e). Accompanied by such a double-switching phenomenon, we uncovered that the *T*_C_ of this (FGT/CS)_3_ superlattice was raised to 206.3 ± 1.6 K (calculated by Arrott-plots, inset of Fig. 2f), reasonably close to the *T*_C_ of ∼201 K determined by zero-field-cooled and field-cooled (*ZFC-FC*) curves (Fig. 2f). Therefore, a dramatic enhancement of *T*_C_ over 60 K is achieved when compared to 140.3 K in 4-layer Fe_3_GeTe_2_. The evolutions of the double-switching AHE and *M-H* curves at various temperatures are provided in Supplementary Figs 5–8. Conjointly, the *T*_C_ modulation and double-switching effect are closely related to the interfacial coupling between the ferromagnetic Fe_3_GeTe_2_ and antiferromagnetic CrSb.

**Figure 3.**
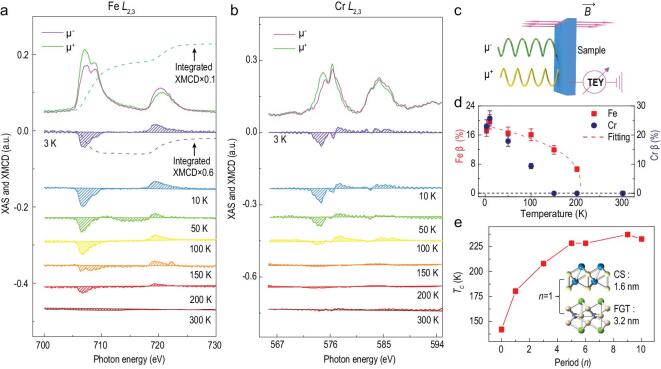
Element-specific magnetic states and *T*_C_ modulation in (FGT/CS)_3_ superlattice. (a) Typical XAS and XMCD spectra of the Fe *L*_2,3_ edge obtained at 3 K. The ferromagnetic state of Fe_3_GeTe_2_ can persist up to 200 K. Dashed lines are the integrations of the spectra, which is used to analyze the moments of Fe (see details in Supplementary section 3). (b) Typical XAS and XMCD spectra of the Cr *L*_2,3_ edge at 3 K. This ferrimagnetic order results from the interfacial CrSb that is converted from the intrinsic antiferromagnetic state, which induces the double-switching behavior in AHE. (c) Experimental setup of the XMCD measurement. Left (μ^−^) and right (μ^+^) circular polarized X-ray incident normally onto the sample surface and in parallel to the magnetic field. (d) Temperature-dependent XMCD percentage of the Fe *L*_3_ and Cr *L*_3_ edge. Here, XMCD percentage (β) is defined in the equation $\beta = \frac{{{\mu ^ - } - {\mu ^ + }}}{{{\mu ^ - }{\rm{ + }}{\mu ^ + }}}$. *T*_C_ = 208.6 ± 7.5 K by fitting the temperature-dependent Fe XMCD percentage using the empirical equation ${(1 - T/{T_C})^\gamma }$ [46,47], consistent with that obtained from the Arrott-plots (206.3±1.6 K). (e) *T*_C_ versus the period *n*, which increases ∼60% from 140.3 ± 2.7 K of the pure 4-layer FGT to 230.9 ± 1.3 K in (FGT/CS)_10_ superlattice. Note that the thicknesses of Fe_3_GeTe_2_ and CrSb are ∼3.2 nm (4-layer) and ∼1.6 nm, respectively.

We further conducted element-specific XMCD at Fe and Cr *L*_2,3_ absorption edges to probe the local electronic character and investigate how the proximity interfacial interaction evolves in (FGT/CS)_3_. In the XMCD measurement, circularly polarized X-ray with 100% left and right polarization was used, denoted as μ^−^ and μ^+^, respectively. XMCD is defined as the difference of the X-ray absorption spectroscopy (XAS), written as the equation $XMCD = {\mu ^ - } - {\mu ^ + }$. Figure 3a presents a typical pair of XAS and XMCD spectra of Fe *L*_2,3_ edge obtained using total electron yield detection mode. The XAS spectra, in good agreement with Fe_3_GeTe_2_ bulks [45] in the spectrum shape and energy positions, confirm that the Fe-magnetism originates from the Fe_3_GeTe_2_ region. Consistent with the *T*_C_ determined by the AHE measurements (206.3 K), the XMCD signals can be distinguished at 200 K and vanish at 300 K (Fig. 3a). Significantly, Cr *L*_2,3_ spectra give a strong XMCD dichroism at 3 K (Fig. 3b), indicating the newly developed Cr magnetic state at the interface. This Cr ferrimagnetic order can be detected at 50 K and becomes much weaker when approaching 100 K (Fig. 3b). By revisiting the AHE measurements, we note that the two magnetic states from intrinsic Fe_3_GeTe_2_ and interfacial CrSb can individually contribute to the resistance jump at each *H*_C_, and accordingly, the double-switching behavior occurs. Here, XMCD percentage (β), defined by $\beta = \frac{{{\mu ^ - } - {\mu ^ + }}}{{{\mu ^ - }{\rm{ + }}{\mu ^ + }}}$, is used to analyze the ferromagnetism. By fitting the temperature-dependent Fe XMCD percentage to the empirical function of ${(1 - T/{T_C})^\gamma }$ [46,47], *T*_C_ is calculated to be 208.6 ± 7.5 K (Fig. 3d), which is consistent with the magneto-transport measurements. The same positive trends of Cr and Fe spectra as a function of the magnetic field indicate parallel interfacial ferromagnetic coupling between Fe_3_GeTe_2_ and CrSb (see details in Supplementary Fig. 10) [47], which agrees with the negative *H*_EX_ at minor loop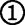 (Fig. 2d).

Now, we summarize the various *T*_C_ for (FGT/CS)_n_ superlattices as a function of period *n*. Here, *T*_C_ is extracted by Arrott-plots (Supplementary Figs 11–12). As shown in Fig. 3e, a giant improvement of *T*_C_ is observed in the (FGT/CS)_n_ superlattice. Once the FGT/CS bilayer is established, denoted as (FGT/CS)_1_, *T*_C_ can be noticeably raised to 178.7 ± 2.5 K, ∼40 K higher than that of the pure 4-layer Fe_3_GeTe_2_. *T*_C_ increases continuously as *n* increases to above 5, above which it saturates at ∼230 K. Despite the dimensionality effect on the *T*_C_ of pure Fe_3_GeTe_2_, which is given in detail in Fig. 1f, when comparing the *T*_C_ in pure 4-layer FGT with (4-layer FGT/CS)_1_, and *T*_C_ in bulk FGT with (4-layer FGT/CS)_9_ where the thickness of FGT in the superlattices is fixed at 4-layer, we believe that besides the dimensionality effect, the FGT/CS interfacial interactions play a more important role in the *T*_C_ increase in these superlattices. The mechanism is proposed in the following section.

## THEORETICAL CALCULATION

**Figure 4.**
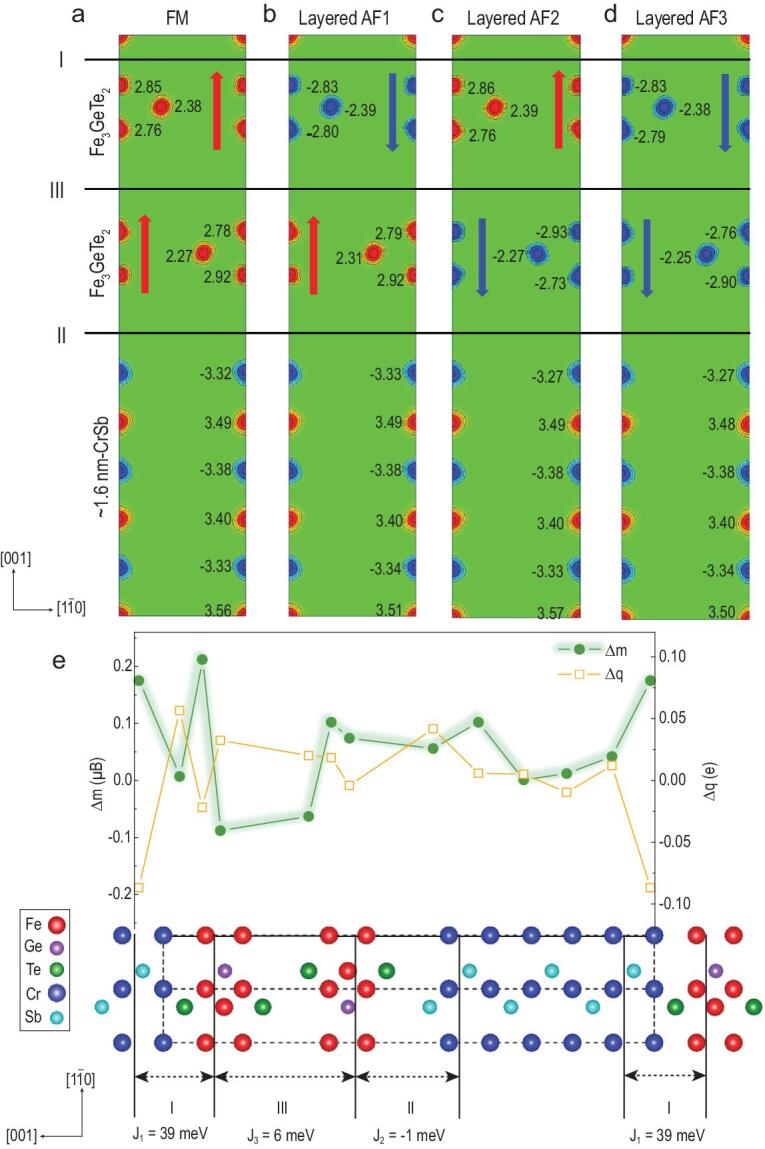
DFT calculations for FGT/CS superlattice. (a–d) Spin density plots in the (110) plane of FGT/CS superlattice in four different magnetic states of FM, Layered AF1, Layered AF2 and Layered AF3, respectively. Red (blue) color stands for the Fe or Cr up (down) spin. The magnetic moments are marked for each atom. (e) Three kinds of interfaces in the FGT/CS superlattice: Fe-Te/Cr-Sb interface named interface I, Fe-Te/Sb-Cr interface named interface II and FGT van der Waals monolayer interface named interface III with the exchange constants for each corresponding interface defined as J_1_, J_2_ and J_3_, respectively. The changes of atomic charge (Δq) and magnetic moments (Δm) of the FGT/CS superlattice against the FGT monolayer and CS bulk. Compared to interface II, larger (Δq, Δm) and the exchange constant J_1_ = 39 meV can be determined at the FM coupled interface I, which result in significant *T*_C_ enhancement in the FGT layer.

To seek the origin of such a *T*_C_ enhancement in the FGT/CS superlattices, we performed DFT calculations within the Generalized Gradient Approximation (GGA) plus U framework. Taking into account the robust FM ground state of Fe_3_GeTe_2_ monolayer and the A-AF state of CS (Supplementary section 5), we constructed four different magnetic states (Fig. 4a–d) for the FGT/CS superlattice to address three magnetic couplings at the Fe-Te/Cr-Sb interface (I), at the Fe-Te/Sb-Cr interface (II) and in-between the two FGT van der Waals (vdW) layers (III), corresponding to the exchange constants J_1_, J_2_ and J_3_, respectively (Fig. 4e). We mapped the calculated energy differences (summarized in Table 1) onto a simple (J_1_, J_2_, J_3_) magnetic exchange model. We found that while the Fe-Te/Cr-Sb interface has a strong FM coupling (J_1_ = 39 meV), the Fe-Te/Sb-Cr interface is very weakly AF coupled (J_2_ = −1 meV), and the two FGT vdW layers have moderate FM coupling (J_3_ = 6 meV). The very weak J_2_ coupling is a result of large Fe−Cr separation by Te−Sb atoms. The strong J_1_ exchange is associated with the intact Fe−Te−Cr pathway in which the Cr atom moves closely to Te to remove its otherwise dangling bond. The moderate J_3_ value of 6 meV here is comparable to the calculated value of 7.5 meV for the FGT bulk, where the experimental vdW interlayer Te−Te distance of 2.94 Å is smaller than the optimized theoretical value of 3.02 Å. Therefore, we suggest that the FGT/CS superlattice has a strong FM Fe-Te/Cr-Sb interface (I) but a weak AF Fe-Te/Sb-Te interface (II) and moderately FM coupled vdW FGT monolayers (III).

**Table 1.** (J_1_, J_2_, J_3_) magnetic exchange model, relative total energy *ΔE* (meV/cell) and total magnetic moments (Tot, μ_B_/fu) of the FGT/CS.

FGT/CS	Energy^a^	Δ$E$ (meV/fu)	Tot (μ_B_/fu)
FM	−J_1_−J_3_+J_2_	0	7.89
Layered-AF1	J_1_+J_3_+J_2_	90	0.30
Layered-AF2	−J_1_+J_3_−J_2_	14	0.46
Layered-AF3	J_1_−J_3_−J_2_	80	$-$ 7.16

^a^J_1_ = 39 meV, J_2_ = −1 meV, J_3_ = 6 meV are derived.

As the Fe-Te/Cr-Sb interface I is strongly FM coupled, it tunes the magnetic behavior of the interfacial FGT monolayer: when this FGT monolayer is changed from the FM ground state to the tri-layered AF state (i.e. up-up-up spins to up-down-up spins as shown in Supplementary Fig. 14c–d, respectively), the total energy rises drastically, from 595 meV/fu for a bare FGT monolayer to 820 meV/fu (per fu of FGT). In contrast, the corresponding energy difference is reduced to 424 meV/fu for the FGT monolayer lying at the Fe-Te/Sb-Cr interface II, which is weakly AF coupled. Obviously, the significant enhancement of FM coupling in the FGT vdW layer at the Fe-Te/Cr-Sb interface I dominates over the reduction at the Fe-Te/Sb-Cr interface II (caused by negative effects from the tensile strain (see Supplementary section 5) and the weak AF interfacial coupling here). Moreover, it is believed that for the interior FGT vdW layers in the FGT/CS superlattice, their intralayer and interlayer FM couplings should be very similar to their bulk cases.

## CONCLUSION

In Fig. 4e, we plot the changes of atomic charge (Δq) and magnetic moments (Δm) of a representative 2-layer FGT/1.6nm-CS superlattice against the FGT monolayer and the CS bulk. It can be observed that the Fe-Te/Cr-Sb interface I has much larger charge/moment changes than those at the Fe-Te/Sb-Cr interface II. More specifically, for the ‘more important’ Fe-Te/Cr-Sb interface I, the Cr atoms donate some electrons to the neighboring FGT vdW layer, and therefore the Cr atoms and FGT monolayer both have increased magnetic moments. Together, these contribute to the above significant enhancement of FM coupling in the FGT vdW layer at the strongly FM coupled Fe-Te/Cr-Sb interface I, and eventually to the remarkable *T*_C_ enhancement in FGT films of the FGT/CS superlattice. Moreover, because of this spin-polarized charge transfer, the interlayer AF coupled Cr layers in CrSb become ferrimagnetic and thus have a net magnetic moment, which accounts for the above XMCD observations (Fig. 3c).

**Figure 5.**
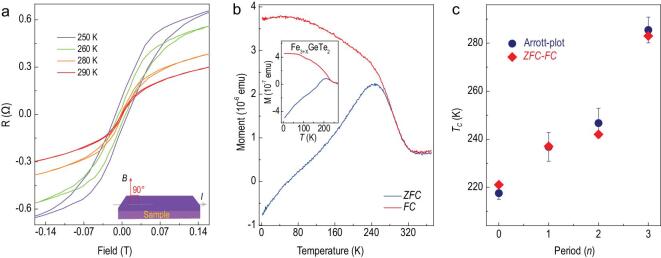
*T*_C_ enhancement in the (Fe_3+x_GeTe_2_/CrSb) superlattice. (a) Temperature-dependent AHE in (Fe_3+x_GeTe_2_/CrSb)_3_. Up to 280 K, hysteresis can still be observed. The inset is the perpendicular geometry for the measurement. (b) *ZFC-FC* curves for (Fe_3+x_GeTe_2_/CrSb)_3_. *T*_C_ can be roughly determined to be ∼280 K, complying with that of 286.7 ± 5.4 K calculated by the Arrott-plots (Supplementary Fig. 13). The inset is the *ZFC-FC* curve for the 4-layer Fe_3+x_GeTe_2_ with *T*_C_ at ∼220 K. (c) Period-dependent Curie temperature. As the period increases, *T*_C_ can be raised from 217.5 ± 2.6 K (*n* = 0, the pure Fe_3+x_GeTe_2_) to 286.7±5.4 K (*n* = 3, the superlattice). The definition of period *n* = 1 is a bilayer structure of Fe_3+x_GeTe_2_ and CrSb, the same as that depicted in Fig. 3d inset. The thickness of Fe_3+x_GeTe_2_ and CrSb is ∼3.2 nm and ∼1.6 nm, respectively.

Inspired by the *T*_C_ tunability in Fe_3+x_GeTe_2_ via chemical doping [9,26], we created a similar superlattice using Fe-rich Fe_3+x_GeTe_2_ with CrSb to achieve even higher *T*_C_. From the AHE measurements, the hysteresis can be distinguished up to 280 K (Fig. 5a), based on which *T*_C_ in (Fe_3+x_GeTe_2_/CrSb)_3_ is calculated to be 286.7 ± 5.4 K (Arrott-plots, Supplementary Fig. 13), in good agreement with the *T*_C_ of ∼280 K determined from *ZFC-FC* (Fig. 5b). Considering the evolutions of the *T*_C_ in the (FGT/CS)_n_ superlattice, in these (Fe_3+x_GeTe_2_/CrSb)_n_ samples, we plotted the *T*_C_ as a function of *n* from *n* = 0 to *n* = 3 in Fig. 5c. Similar to the period-dependent *T*_C_ in the (FGT/CS)_n_ superlattice, with the period increasing, *T*_C_ shows a rising trend up to *n* = 3, with ∼70 K increase to 286.7 K ± 5.4 K (*n* = 3). To this point, we achieved a *T*_C_ of ∼286.7 K in 4-layer 2D Fe_3+x_GeTe_2_ films via the proximity effect.

In summary, we have developed atomically thin 2D ferromagnetic Fe_3_GeTe_2_ films on a large scale even down to bilayer by precisely controlling epitaxial growth rate. Combined with the AF CrSb, a parallel ferromagnetic interface interaction between Fe_3_GeTe_2_ and CrSb induces an enormous *T*_C_ enhancement up to 286.7 K in the superlattice structure from a low-*T*_C_ of 140.3 K in the pure 4-layer Fe_3_GeTe_2_. Interestingly, the double-switching behavior is observed for the first time in this system as a result of a proximity effect between FGT and Cr layers. In support of these abundant experiments, our DFT calculations found that the interfacial Cr layers retained their interlayer AF coupling but had a net FM magnetic moment, and that doping the spin-polarized electrons via the interfacial Cr layer gives rise to the *T*_C_ enhancement of the Fe_3_GeTe_2_ films. Our approach of feasible modulation of *T*_C_ enabled by the FM/AF proximity effect, together with the capability of wafer-scale growth, provides a realistic platform for spintronic devices based on 2D FMs.

## METHODS

### Thin-film synthesis

Thin films were grown on mica and (0001) sapphire in a Perkin Elmer 430 MBE system with a base vacuum of 2.5 × 10^−9 ^Torr. The growth substrate temperature for Fe_3_GeTe_2_ was ∼310°C, with the source temperatures of Fe (99.99%), Ge (99.999%) and Te (99.999%) at 1165°C, 1020°C and 285°C, respectively. They were co-evaporated from standard Knudsen cells. CrSb films were grown at the substrate temperature of 280°C, with Cr (99.99%) and Sb (99.999%) cell temperatures of 1180°C and 400°C, respectively. The flux of each element was calibrated by the crystal monitor. The MBE system was equipped with an *in situ* RHEED.

### Thin-film characterizations

Structural characterizations of Fe_3_GeTe_2_ and CrSb samples were carried out by X-ray diffraction (Bruker D8 Discover, Bruker Inc., Billerica, MA, USA) and TEM (FEI Tecnai F20) equipped with EDS. Sample composition and doping concentration were determined by EDS. Cross-section TEM samples were prepared by Focused ion beam (FEI Scios DualBeam).

### Electrical and magnetization characterizations

Magneto-transport measurements were performed with the Physical Properties Measurement System by Quantum Design. The magneto-transport devices were confined to the Hall-bar geometry. Experimental data were collected using lock-in amplifiers (Stanford Research 830, Stanford Research Systems, Sunnyvale, CA, USA). The magnetization measurements were taken using DC-Superconducting-Quantum-Interface-Devices (SQUID) by Quantum Design.

### X-ray magnetic circular dichroism measurement

XMCD measurements were performed on Beamline I10 at the Diamond Light Source, UK (100% polarized X-rays), and beamline 6.3.1 at the Advanced Light Source, Berkeley, CA (65% polarized X-rays). During the data acquisition, the polarization of X-ray is switched with a fixed magnetic field at every energy point (Beamline I10), and the field direction is switched without changing the polarization at every energy point (Beamline 6.3.1). Such polarization switching at each energy point ensures identical sample conditions for the measurements.

### Density functional theory calculations

We performed DFT calculations using the Vienna Ab initio Simulation Package with a plane wave basis set [48]. The ionic potentials including the effect of core electrons are described by the projector augmented wave method, and the GGA was used as the exchange-correlation functional [49]. To better describe the interactions between CrSb and Fe_3_GeTe_2_ in the superlattices, the vdW corrections were considered within Grimme's approach (DFT-D2) [50]. The plane waves with the kinetic energy up to 400 eV were employed to expand the electronic wave functions. Integration over the first Brillouin zone was carried out using the Monkhorst-Pack grid of 7 × 7 × 5 k-point mesh. The structural relaxations were performed till the Hellmann-Feynman force on each atom was smaller than 0.01 eV/Å. Experimental lattice constants were adopted [34,36]. A 24-atom superlattice consisting of 1 × 1 × 1 unit cell of Fe_3_GeTe_2_ (12 atoms) and 1 × 1 × 3 lattice of CrSb (12 atoms) was used to study the interfacial interactions. The Coulomb and exchange parameters U = 3.5 (3.0) eV and J = 0.9 (0.9) eV were chosen for Fe (Cr) 3d electrons [51,52]. Bader charge analysis was used to identify the interfacial Cr-Fe charge transfer [53].

## Supplementary Material

nwz205_Supplemental_File
