# Supplementary material for: Two-dimensional ferromagnetic superlattices
Source: Natl Sci Rev. 2019 Dec 16;7(4):745–54. doi: 10.1093/nsr/nwz205 (PMC8289050; doi:10.1093/nsr/nwz205)
Supplement: nwz205_Supplemental_File [file nwz205_supplemental_file.docx]

**Supplementary information for**

**Two-dimensional Ferromagnetic Superlattices**

Shanshan Liu,^1,2#^ Ke Yang,^1,3#^ Wenqing Liu,^4#^ Enze Zhang,^1,2^ Zihan Li,^1,2^ Xiaoqian Zhang,^5^ Zhiming Liao,^6^ Wen Zhang,^7^ Jiabao Sun,^4^ Yunkun Yang,^1,2^ Han Gao,^6^ Ce Huang,^1,2^ Linfeng Ai,^1,2^ Ping Kwan Johnny Wong,^8^ Andrew Thye Shen Wee,^7,8^ Alpha T. N’Diaye,^9^ Simon A. Morton,^9^ Xufeng Kou,^10^ Jin Zou,^6,11^ Yongbing Xu,^5^ Hua Wu,^1,3,12*^ Faxian Xiu^1,2,1^^2^**^*^**

^1^State Key Laboratory of Surface Physics and Department of Physics, Fudan University, Shanghai 200433, China

^2^Institute for Nanoelectronic Devices and Quantum Computing, Fudan University, Shanghai 200433, China

^3^Laboratory for Computational Physical Sciences (MOE), Fudan University, Shanghai 200433, China

^4^Department of Electronic Engineering, Royal Holloway University of London, Egham TW20 0EX, United Kingdom

^5^School of Electronic Science and Engineering, Nanjing University, Nanjing 210093, China

^6^Materials Engineering, The University of Queensland, Brisbane QLD 4072, Australia

^7^Department of Physics, National University of Singapore, 2 Science Drive 3, Singapore 117542, Singapore

^8^Centre for Advanced 2D Materials and Graphene Research Centre, National University of Singapore, 6 Science Drive 2, Singapore 117546, Singapore

^9^Lawrence Berkeley National Laboratory, Berkeley, California 94720, United States

^10^School of Information Science and Technology, ShanghaiTech University, Shanghai 201210, China

^11^Centre for Microscopy and Microanalysis, The University of Queensland, Brisbane QLD 4072, Australia

^12^Collaborative Innovation Center of Advanced Microstructures, Nanjing 210093, China

^#^ These authors contribute equally to this work.

*^*^*Correspondence and requests for materials should be addressed to F. X. (E-mail: [Faxian@fudan.edu.cn](mailto:Faxian@fudan.edu.cn)), H.W. ([wuh@fudan.edu.cn](mailto:wuh@fudan.edu.cn)).

**Content:**

1. **Sample growth and characterizations**
2. **Double switching behavior in (FGT/CS)_n_ superlattices**
3. **XMCD in (FGT/CS)_3_ superlattice**
4. **Period-dependent** **Curie Temperature in (FGT/CS)_n_ superlattices**
5. **Density functional theory calculations on FGT/CS heterostructure**
6. **Reference**

**1:**  **Sample growth and characterizations**

As displayed in Supplementary Fig. 1, streaky RHEED patterns for (FGT/CS)_3_ superlattice indicate a smooth surface for each layer growth, which guarantees a sharp interface between Fe_3_GeTe_2_ and CrSb. High crystalline {002}-Fe_3_GeTe_2_ and {002}-CrSb films were further proved by XRD measurements (Supplementary Fig. 2). The magnetic property of Fe_3_GeTe_2_ thin films was measured by AHE in a Hall-bar geometry. Generally, the Hall resistance (*R*_xy_) for ferromagnetic materials can be written as$R_{xy}=R_{H}B+R_{AH}M$, which composes of the normal Hall contribution (*R*_H_*B*) and magnetization contribution (*R*_AH_*M*). Subtracting the normal Hall part from the raw data yields the AHE component which is dependent on the angle between *B* and the normal vector of the sample surface (Supplementary Fig. 3a). Obviously, even down to 4-layer, the easy axis of Fe_3_GeTe_2_ is still along *c*-axis (out-of-plane) as *H*_C_ increases with the angle switching from 0 ̊ to 90 ̊ (Supplementary Fig. 3a inset). To estimate *T*_C_ precisely, the Arrott-plot method was employed (Supplementary Fig. 3b inset). In the Arrott-plot, the ratio of (*R*_xy_/*R*_xx_^n^) can be used to track the magnetization in the metallic materials[1], while the (*R*_xy_/*R*_xx_^n^)^2^ is plotted against *B*/(*R*_xy_/*R*_xx_^n^), the intercept to the y-axis is positive (negative) for ferromagnetic (paramagnetic) state, and then *T*_C_ can be determined when the intercept goes to zero. Note that n=2 corresponds to AHE contributed mainly by intrinsic Berry-phase and n=1 for the skew scattering[1,2]. Consistent with the 5% change in the *R*_xx_ reported by Zhe Wang, et.al[3], we have also observed a similar behavior of *R*_xx_ in our thin films. In the recent work[4], the AHE in Fe_3_GeTe_2_ is dominated by the intrinsic contribution. Therefore, we have chosen n=2 in the Arrott-plot process and the little change in *R*_xx_ suggests that n=1 or 2 has negligilible effect on the *T*_C_ determination[1]. *T*_C_ of 4-layer FGT is determined to be ~140.3±2.7 K, much lower than that of bulk[5] (~220 K). *Zero-field-cooled* and *field-cooled* (*ZFC-FC*) curves under 200 Oe magnetic field of 4-layer FGT are displayed in Supplementary Fig. 3b. The ferromagnetic order in CrSb can be excluded by measuring *M-H* curves at two measurement geometries including in-plane and out-of-plane (Supplementary Fig. 4).

**Supplementary Figure 1. RHEED patterns of (Fe_3_GeTe_2_/CrSb)_3_**. Streaky RHEED patterns suggest a smooth surface.

**Supplementary Figure 2. XRD results of (a)** **Fe_3_GeTe_2_ and (b) CrSb**. By comparing Fe_3_GeTe_2_ with PDF Card# 01-075-5620 and CrSb with PDF Card#96-900-8890, Fe_3_GeTe_2_ is determined to be the hexagonal structure with the space group P6_3_/mmc, and CrSb is NiAs-type, possessing the antiferromagnetic property with a Neel temperature *T*_N_ ~ 710 K (bulk)[6,7].

**Supplementary Figure 3. Magnetic properties of 4-layer Fe_3_GeTe_2_ film**. (a) Angle-dependent AHE at 2.5 K with the angle definition in the bottom inset. The easy axis is determined to be out-of-plane, as *H*_C_ increases correspondingly with θ tilting from 0 ̊ to 90 ̊. (b) *ZFC*-*FC* curves under 200 Oe. With the temperature decreasing, the magnetization in *FC* increases, displaying an opposite trend to that in *Z**FC*. *T*_C_ is determined to be 140.3±2.7 K calculated by Arrott-plots as displayed in the inset. Dashed lines represent the linear fits. (c) Temperature-dependent *M-H* curves. The *M-H* curve at 125 K displays a quasi-linear behavior (inset).

**Supplementary Figure 4. *M-H* results for the pure CrSb film**. (a) *M-H* curve under in-plane geometry. (b) Zoom-in view in the low field region. (c) and (d), *M-H* curve under perpendicular magnetic field. As expected, the *M-H* curves do not display hysteresis under both in-plane and out-of-plane geometries.

**2:** **Double switching behavior in (FGT/CS)_n_ superlattice**

Temperature-dependent double-switching behavior in AHE and *M-H* curves is presented in Supplementary Fig. 5-6 and is summarized in Supplementary Fig. 7. As the superlattice period increases from n=1 to 10, the double-switching becomes more evident. This period-dependent double-switching phenomenon can be also characterized by *H*_EX_ since a strong interface coupling induces a large *H*_EX_. With the temperature rising, *H*_EX_ decreases monotonously, indicating gradually-weakened interface interactions, which is consistent with the temperature-dependent AHE. With increasing periods, however, *H*_EX_ increases as the interface contribution becomes more significant. However, under the condition of the fixed periods, with decreasing the Fe_3_GeTe_2_ thickness, the contribution from the ferromagnetic interface CrSb will play a more prominent role and then the induced double-switching will be sharper (Supplementary Fig. 8).

To detect whether the exchange-bias effect exists in (FGT/CS)_3_ or not, we cooled down the sample temperature to 2.5 K with the applied positive (negative) magnetic field of 9 T (–9 T) and then measured the AHE. As displayed in Supplementary Fig. 9, there is no evident sign of exchange bias in this system, which is possibly due to the higher *T*_N_ of CrSb (~710 K) than *T*_C_ of Fe_3_GeTe_2_ (~140 K)[8,9].

**Supplementary Figure 5.** Temperature-dependent double switching behavior in (FGT/CS)_1_ (a-e), (FGT/CS)_3_ (f-j), and (FGT/CS)_10_ (k-o). AHE curves with one complete major loop and two minor loops at 25 K, 10 K, 7 K, 5 K, and 2.5 K are displayed. Positions of exchange field *H*_EX_ are marked in black dashed lines. With the temperature increasing, the double switching behavior becomes weaker and *H*_EX_ decreases continuously.

**Supplementary Figure 6.** Double switching behavior in *M-H* hysteresis of (FGT/CS)_3_ superlattice at the temperature of (a) 10 K, (b) 35 K, and (c) 50 K. (d) Temperature-dependent *M-H* hysteresis in the range of 70~150 K. Above 50 K, the double switching phenomenon vanishes.

**Supplementary Figure 7. Summary of temperature-dependent *H*_EX_**. (a) Periods-dependent double-switching. By increasing periods, the double-switching properties become more evident, suggesting the enhanced interface coupling effect. n=1 is defined as a period of 4-layer Fe_3_GeTe_2_ coupled with ~1.6 nm-CrSb, illustrated as a sketch in the inset. (b) Period-dependent *H*_EX_ extracted from the double-switching AHE.

**Supplementary Figure 8. The AHE double switching behavior in (FGT/CS)_n_ superlattices**. Under the condition of the fixed Fe_3_GeTe_2_ thickness, the minor switching features at lower magnetic fields become stronger and broader as the period increases. Once the period of the superlattice is fixed, this switching feature will be sharper and more evident in a superlattice with thinner Fe_3_GeTe_2_.

**Supplementary Figure 9. Exchange-bias measurements under the out-of-plane geometry for (FGT/CS)_3_ superlattice**. To verify the exchange bias effect, the AHE experiments under the condition of +9T, 0T, -9T magnetic field cooling were carried out at 40 K, 100 K, and 135 K as shown in (a), (d), and (g), respectively. No visible exchange bias has been observed under these measurement conditions possibly due to the lower *T*_C_ of Fe_3_GeTe_2_ than the *T*_N_ of CrSb[8,9]. (b), (c), (e), (f), and (h) are the zoom-in views to check the exchange bias at different temperatures.

**3:**  **XMCD in (FGT/CS)_3_ superlattice**

The XMCD spectra are displayed in Fig. 3 of the main text. The spin (m_s_) and orbital (m_l_) magnetic moment can be obtained by applying sum rules[10,11], according to the equations,

$\begin{matrix} \text{m}_{l}=-\frac{4}{3}n_{h}\frac{\int_{L2,3} (\mu^{-}-\mu^{+})dE}{\int_{L2,3} (\mu^{-}+\mu^{+})dE} \\ \text{m}_{s}=-n_{h}\frac{6\int_{L3} (\mu^{-}-\mu^{+})dE-4\int_{L2,3} (\mu^{-}-\mu^{+})dE}{\int_{L2,3} (\mu^{-}+\mu^{+})dE}\times SC-\left\langle T_{Z}\right\rangle\end{matrix}$ ,

where n_h_, E, SC, and <T_Z_>, stand for the number of 3d holes, photon energy, spin correction factor, and magnetic dipole term, respectively. Here, assuming n_h_=4 and SC=1 that is employed in the XMCD measurements for Fe_3_GeTe_2_ bulk[12] and we neglect the small <T_Z_> that gives an error ~5%[13], m_s_ at 3 K and 150 K are calculated to be ~1.05 and 0.42 μ_B_/Fe, respectively, smaller than the value of 1.48 μ_B_/Fe estimated in the Fe_3_GerTe_2_ bulk[12].

The CrSb coupling effect on the enhanced ferromagnetic order in Fe_3_GeTe_2_ can be generally addressed using the interfacial XMCD-intensity model proposed by Maccherozzi *et. al.* for the dilute magnetic semiconductors[14]. The XMCD intensity measured by the TEY mode is commonly in an exponentially decay with the penetration distance, which can be written as[14,15]

$$\text{Fe }XMCD=\frac{\int_{0}^{\infty} \delta(\mathrm{x})\rho(\mathrm{x})\mathrm{e}^{-x/\lambda_{e}}dx}{\int_{0}^{\infty} \rho(\mathrm{x})\mathrm{e}^{-x/\lambda_{e}}dx}$$

where λ_e_ is the mean electron escape length. Based on the XMCD intensity integration function and provided the following conditions: (i) sharp interface (Fig. 1d-e); (ii) uniform distribution of Fe; and (iii) step-like dichroism versus thickness d, where δ(x)= δ_exp_ for x<d_min_ and δ(x)=0 elsewhere. Here d_min_ limits the interfacial Fe_3_GeTe_2_ thickness that contributes to the XMCD signal above 140 K (intrinsic *T*_C_ of 4-layer Fe_3_GeTe_2_), and then correspondingly d_min_ can be described by$\text{d}_{\min}=d_{FGT}+\lambda_{e}\ln\left[\frac{\delta_{\exp}}{\delta_{sat}}+(1-\frac{\delta_{\exp}}{\delta_{sat}})\mathrm{e}^{-d_{FGT}/\lambda_{e}}\right]$[16]. Roughly quoting m_s_= 1.05 μ_B_/Fe (3 K) and 0.42 μ_B_/Fe (150 K), and λ_e_=5 nm, we obtain d_min_=1.5 nm at 150 K.

Field-dependent XMCD measurements were performed at a fixed temperature of 3 K. Displayed in Supplementary Fig. 10, the Fe and Cr XMCD percentages increase initially as the magnetic field rises and then saturate, similar to the AHE and *M-H* results. Compared with the field-dependent Fe-XMCD percentage, the evolution of Cr-*L*_3_ is almost the same as that of Fe-*L*_3_ edge, further confirming a ferromagnetic coupling[17,18] between the magnetic moments of Cr and Fe which is also consistent with the negative *H*_EX_ at minor loop**①** in the double-switching AHE results (Fig. 2d).

**Supplementary Figure 10. The evolution of XMCD percentage in (FGT/CS)_3_.** Field-dependent XMCD percentage obtained at Fe *L*_3_ edge and Cr *L*_3_ edge at a fixed temperature of 3 K. The similar trend of Fe and Cr XMCD percentage versus magnetic field suggests an interfacial ferromagnetic coupling[17,18] between Fe and Cr atoms.

**4: Period-dependent Curie Temperature in (FGT/CS)_n_ superlattices**

**Supplementary Figure 11. Arrott-plots of (FGT/CS)_n_ superlattices**. The thickness of Fe_3_GeTe_2_ and CrSb is ~3.2 nm (4-layer) and ~1.6 nm, respectively. The *T*_C_ of each superlattice is extracted.

**Supplementary Figure 12. Arrott-plots of (FGT/CS)_n_ superlattices**. The thickness of Fe_3_GeTe_2_ and CrSb is ~1.6 nm (2-layer) and ~1.6 nm, respectively. The *T*_C_ of each superlattice is extracted.

**Supplementary Figure 13. Arrott-plots of (Fe_3+x_GeTe_2_/CrSb)_n_ superlattices**. *T*_C_ is determined to be (a) 217.5±2.6 K, (b) 236.8±6.0 K, (c) 246.7±6.2 K, (d) 286.7±5.4 K, respectively. (e) and (f) are the *ZFC-FC* curves. The thickness of Fe_3+x_GeTe_2_ and CrSb is ~3.2 nm (4-layer) and ~1.6 nm, respectively.

**5: Density functional theory calculations on FGT/CS heterostructure**

We performed DFT calculations for FGT monolayers, CS bulk, and the FGT/CS superlattice. The cell of the superlattice is modeled by 1 unit cell of FGT (2 van der Waals monolayers) plus 3 unit cells of CS (6 Cr-Sb layers) with two different interfaces: Fe-Te/Cr-Sb and Fe-Te/Sb-Cr. Our GGA calculations with lattice optimization show that the FGT monolayer has the lattice constants *a*=*b*=4.006 Å, being almost the same as the experimental bulk value of *a*=*b*=3.991 Å (Supplementary Table 1)[19]. Correspondingly, the optimized lattice constants of the bulk CrSb, *a*=*b*=4.180 Å and *c*=5.351 Å, are close to the experimental ones *a*=*b*=4.108 Å and *c*=5.440 Å within the error of 2%[20]. Therefore, in the calculations for the FGT/CS superlattice, we have fixed the planar lattice constants at the experimental *a*=*b*=4.108 Å of the bulk CrSb, and have optimized the *c*-axis parameter and the atomic positions using the GGA+vdW (van der Waals) functionals. In this sense, the FGT monolayers are under a 2.9% bi-axial tensile strain.
 To set up a reference, we first calculated the strength of the magnetic coupling in FGT monolayer and CS bulk, respectively. Our GGA+U calculations show that the AF ground state of CS bulk is more stable than the FM state by 224 meV/fu with the states defined in Supplementary Fig.14a-b, and that the FM ground state of the FGT monolayer with tri-layer Fe is much more stable than the tri-layered AF state by 595 meV/fu with the states defined in Supplementary Fig.14c-d (Supplementary Table 2). Both the large values imply that their respective magnetic ground states would remain robust despite the formation of the FGT/CS superlattice. In addition, our calculations find that when the FGT monolayer is under a 2.9% bi-axial tensile strain, its FM stability against the tri-layered AF state is reduced from 595 meV/fu to 445 meV/fu (Supplementary Table 2). This result shows that the itinerant FM behavior of Fe_3_GeTe_2_ is weakened by the increasing atomic distance upon the tensile strain. Therefore, the tensile strain would disfavor the *T*_C_ enhancement of the FGT films in the FGT/CS superlattice.

**Supplementary Table 1**. The experimental and optimized lattice constant of bulk CrSb and monolayer Fe_3_GeTe_2_.

**Supplementary Table 2**. Relative total energy of the bulk CrSb and monolayer Fe_3_GeTe_2_ under different lattice constant.

**Supplementary Figure 14.** Magnetic states of FM and A-AF in bulk CrSb are illustrated in (a) and (b). (c) and (d) are the Magnetic states of FM and Tri-layered AF in monolayer Fe_3_GeTe_2_.

1. **Supplementary Reference:**

1. Stolichnov I, Riester SWE, Trodahl HJ *et al.* Non-volatile ferroelectric control of ferromagnetism in (Ga,Mn)As. *Nat Mater* 2008;**7**:464–7.

2. Jungwirth T, Niu Q, MacDonald AH. Anomalous Hall Effect in Ferromagnetic Semiconductors. *Phys Rev Lett* 2002;**88**:207208.

3. Wang Z, Sapkota D, Taniguchi T *et al.* Tunneling Spin Valves Based on Fe_3_GeTe_2_/hBN/Fe_3_GeTe_2_ van der Waals Heterostructures. *Nano Lett* 2018;**18**:4303–8.

4. Wang Y, Xian C, Wang J *et al.* Anisotropic anomalous Hall effect in triangular itinerant ferromagnet Fe_3_GeTe_2_. *Phys Rev B* 2017;**96**:134428.

5. Chen B, Yang J, Wang H *et al.* Magnetic Properties of Layered Itinerant Electron Ferromagnet Fe_3_GeTe_2_. *J Phys Soc Jpn* 2013;**82**:124711.

6. He QL, Kou X, Grutter AJ *et al.* Tailoring Exchange Couplings in Magnetic Topological Insulator/Antiferromagnet Heterostructures. *Nat Mater* 2016;**16**:94–100.

7. Takei WJ, Cox DE, Shirane G. Magnetic Structures in the MnSb-CrSb System. *Phys Rev* 1963;**129**:2008–18.

8. Nogués J, Schuller IK. Exchange bias. *J Magn Magn Mater* 1999;**192**:203–32.

9. Wang F, Xiao D, Yuan W *et al.* Observation of Interfacial Antiferromagnetic Coupling between Magnetic Topological Insulator and Antiferromagnetic Insulator. *Nano Lett* 2019;**19**:2945–52.

10. Thole BT, Carra P, Sette F *et al.* X-ray circular dichroism as a probe of orbital magnetization. *Phys Rev Lett* 1992;**68**:1943–6.

11. Carra P, Thole BT, Altarelli M *et al.* X-ray circular dichroism and local magnetic fields. *Phys Rev Lett* 1993;**70**:694–7.

12. Zhu J-X, Janoschek M, Chaves DS *et al.* Electronic correlation and magnetism in the ferromagnetic metal Fe3GeTe2. *Phys Rev B* 2016;**93**:144404.

13. Huang DJ, Chang CF, Jeng H-T *et al.* Spin and Orbital Magnetic Moments of Fe_3_O_4_. *Phys Rev Lett* 2004;**93**:077204.

14. Maccherozzi F, Sperl M, Panaccione G *et al.* Evidence for a Magnetic Proximity Effect up to Room Temperature at Fe/(Ga,Mn)As Interfaces. *Phys Rev Lett* 2008;**101**:267201.

15. Frazer BH, Gilbert B, Sonderegger BR *et al.* The probing depth of total electron yield in the sub-keV range: TEY-XAS and X-PEEM. *Surf Sci* 2003;**537**:161–7.

16. Liu W, He L, Xu Y *et al.* Enhancing Magnetic Ordering in Cr-Doped Bi_2_Se_3_ Using High-T_C_ Ferrimagnetic Insulator. *Nano Lett* 2015;**15**:764–9.

17. Ye M, Li W, Zhu S *et al.* Carrier-mediated ferromagnetism in the magnetic topological insulator Cr-doped (Sb,Bi)_2_Te_3_. *Nat Commun* 2015;**6**:8913.

18. Zhang W, Zhang L, Wong PKJ *et al.* Magnetic Transition in Monolayer VSe_2_ via Interface Hybridization. *ACS Nano* 2019;**13**:8997–9004.

19. Deiseroth H-J, Aleksandrov K, Reiner C *et al.* Fe_3_GeTe_2_ and Ni_3_GeTe_2_ – Two New Layered Transition-Metal Compounds: Crystal Structures, HRTEM Investigations, and Magnetic and Electrical Properties. *Eur J Inorg Chem* 2006;**2006**:1561–7.

20. Willis BTM. Crystal structure and antiferromagnetism of CrSb. *Acta Crystallogr* 1953;**6**:425–6.
